# The gendered behaviors displayed by Disney protagonists

**DOI:** 10.3389/fsoc.2024.1338900

**Published:** 2024-05-06

**Authors:** Lucy L. Clarke, Benjamin Hine, Dawn England, Poppy P. M. S. Flew, Ritaj Alzahri, Stepheni N. Juriansz, Ma. J. B. C. Garcia

**Affiliations:** ^1^Department of Psychology, University of West London, London, United Kingdom; ^2^Department of Education, University of Birmingham, Dubai, United Arab Emirates; ^3^Department of Psychology, University of West London, London, United Kingdom; ^4^School of Health and Education, Middlesex University, Dubai, United Arab Emirates; ^5^Department of Psychology, Middlesex University, Dubai, United Arab Emirates

**Keywords:** Disney, gender, princess, prince, gender roles, content analysis, children's media

## Abstract

Previous research suggests that the portrayal of male and female protagonists in Disney animations may be changing over time. The current study examined the portrayal of gendered behaviors displayed within some of Disney's most successful animated feature length films, including those beyond the Disney princess franchise. Extending the scope of the Disney animated films analyzed was important because both young girls and young boys report little personal interest in male characters within the Disney princess animations. This suggests that it is important to look beyond the Disney princess franchise to understand the gendered behaviors displayed by potentially influential male Disney protagonists. The current study also considered a greater number of masculine and feminine behaviors as well as some gender-neutral traits which had yet to be incorporated. A quantitative content analysis of 39 Disney protagonists from films released between 1937 and 2021 was conducted. The results revealed that male and female protagonists were statistically higher in feminine than masculine traits. Female protagonists from the earliest animations were the most feminine. However, there was no statistical difference in the gendered portrayals of females in the animations released in the 1990s and those released from 2009 to 2021 suggesting some continued stereotyping in females' profiles. Alternatively, male characters were more feminine relatively consistently across time-points. This study concludes that Disney is persistently portraying stereotyped female protagonists, and this could have implications on young females' behavioral profiles. However, the extent to which feminine traits are being celebrated when displayed by male protagonists needs to be examined, as well as the potential relationship between such messages and boys' behaviors and children's conceptualizations of gender more broadly.

## 1 Introduction

Several previous studies exploring the behavior of protagonists in Disney animated feature length films have suggested that there are numerous gender stereotypical messages present (Dundes, 2001; Towbin et al., 2004; Giroux and Pollock, 2010; England et al., 2011; Dundes and Streiff, 2016; Streiff and Dundes, 2017a,b; Hine et al., 2018a; Primo, 2018). Problematic messages around race (both independently and in relation to gender) have also been consistently identified in Disney's animated feature films by academics (Towbin et al., 2004; Giroux and Pollock, 2010; England et al., 2011; Streiff and Dundes, 2017a,b; Hine et al., 2018a; Primo, 2018). The proposed impact of such messages is supported by several theoretical perspectives which suggest that problematic gender representations can affect children's understanding of gender, for example, Social Cognitive Theory of Gender Development (Bussey and Bandura, 1999), and Gender Schema Theory (Bem, 1981).

Moreover, the gendered behaviors displayed in Disney animations are likely to be particularly influential because this corporation has been one of the most popular producers of children's animated content for over eight decades, meaning further research in this area is therefore warranted and beneficial. Further, while all animation has a sense of innocence, no other animation studio has situated itself as strongly in this way as Disney (Bell et al., 1995; Giroux, 1995; Wasko, 2001; Wells, 2002; Wynns and Rosenfeld, 2003; Giroux and Pollock, 2010; Sammond, 2019). Disney has transformed often violent or gruesome fairy tales into family-friendly, magical, and heart-warming feature films which is one way that its output has been perceived as “innocent.” However, critics argue that presenting idealistic versions of fairy tales and historical events is dangerous (Towbin et al., 2004; Giroux and Pollock, 2010; Heatwole, 2016). Therefore, although Disney animation has largely maintained its innocent reputation in the domestic and familial sphere, its innocence has been questioned in the academic sphere (Bell et al., 1995; Giroux, 1995; Wasko, 2001; Wells, 2002; Wynns and Rosenfeld, 2003; Giroux and Pollock, 2010; Sammond, 2019).

Studies which have sought to quantify the gendered behavior of Disney protagonists, whilst illuminating, have largely utilized the same coding scheme; a framework that may benefit from review due to significant limitations (Padilla-Walker et al., 2013; Hefner et al., 2017). Additionally, many studies have focused on the Disney princess franchise which was created to enable Disney to successfully market (and thus profit from) the oldest and much-loved Disney animations featuring prince and princess characters (such as *Snow White and the Seven Dwarfs*, Cottrell et al., 1937) alongside the more recent princess releases (such as *The Little Mermaid*, Clements and Musker, 1989). Indeed, by bringing the now 13 princess (Disney Princess, 2024) films together in this way, Disney ensured that even the earliest princess releases stayed relevant and continued to be consumed over 50 years after initial release (Heatwole, 2016). However, this focus has meant that there has been little quantitative investigation of the gendered profiles of other influential Disney protagonists (Holcomb et al., 2014; Azmi et al., 2016), despite important calls to consider a wider repertoire of media content from which children receive influential gendered messages (Ward and Grower, 2020). Further, assessing animated films which lie outside of the female targeted Disney princess franchise will also provide insight into the representation of gender displayed by Disney protagonists that may be more likely to appeal to young boys. For example, those within *The Hunchback of Notre Dame* (Trousdale and Wise, 1996) and *Tarzan* (Buck and Lima, 1999) which each yielded over $300 million globally (Box Office Mojo, 1996, 1999).

The current study, therefore, aims to extend previous work to consider and overcome some of the limitations identified within the previous quantitative content coding studies conducted by England et al. (2011) and Hine et al. (2018a). A quantitative content analysis of 39 Disney protagonists spanning films released from 1937 to 2021 for their levels of stereotypically masculine, feminine and gender-neutral behavior was thus conducted. This study also applied an expanded coding scheme across a broad range of films and protagonists to assess the gendered behavior displayed and to identify the chronological changes in the representation of men and women in Disney feature length animations.

### 1.1 Findings from previous quantitative content analyses

The most recent quantitative content coding analyses of the gendered behaviors displayed in Disney animated feature length films (England et al., 2011; Hine et al., 2018a) have focused exclusively on the Disney princess animations. In these publications, the earliest princesses, those in *Snow White and the Seven Dwarfs* (Cottrell et al., 1937), *Cinderella* (Geronimi et al., 1950), and *Sleeping Beauty* (Clark et al., 1959), were the most feminine of all the princesses in the franchise. They were identified as being highly submissive (England et al., 2011), passive and victimized (Towbin et al., 2004; Whitley, 2013) and domestic work was central to portrayals of femininity in *Snow White and Seven Dwarves* (Cottrell et al., 1937) and *Cinderella* (Geronimi et al., 1950) especially (Whitley, 2013; Heatwole, 2016). This finding is perhaps unsurprising considering the time the films were released, however, it is important to note that they are still likely to be influential to young audiences who remain vulnerable to the stereotypical messages prevalent within them. Curiously, while the female protagonists in the earliest Disney princess animations were highly gender stereotypical, the prince characters within those films were high in both masculine and feminine behavior (England et al., 2011). It is unclear as to why the female characters were highly stereotyped and the male characters were not, although the prince characters in this era of Disney animations lacked screen time (Davis, 2007) which left little behavior to be considered for analysis (England et al., 2011).

Princess films released between 1989 and 1998 (*The Little Mermaid*, Clements and Musker, 1989; *Beauty and the Beast*, Trousdale and Wise, 1991; *Aladdin*, Clements and Musker, 1992; *Pocahontas*, Gabriel and Goldberg, 1995; and *Mulan*, Bancroft and Cook, 1998) referred to as the “middle” princess animations by England et al. (2011) and Hine et al. (2018a), included princesses who were regarded as more independent in comparison to “earlier” princesses (Davis, 2007). The princesses in this era were statistically more masculine than the “earlier” princesses (England et al., 2011). However, these films have been criticized for still taking place within patriarchal societies (Giroux and Pollock, 2010), that are ruled by the princesses' overpowering (albeit caring) fathers (Holcomb et al., 2014). These animations have also been criticized for displaying princesses who are predominately focused on romantic relationships that are largely idealistic and unhealthy (Hefner et al., 2017), much like their earlier counterparts. Indeed, almost all the “middle” princess films end with the princess marrying or being romantically involved, highlighting Disney's reliance on romance to provide “happy endings.” The prince characters in the “middle” movies were slightly more masculine than the “earliest” princes although this difference was not statistically significant (England et al., 2011). Perhaps most notably, the “middle” princes were much more muscular, and many were leaders (England et al., 2011) suggesting some more stereotypical portrayals of masculinity. The “middle” animations are therefore considered to have mixed gendered messages (England et al., 2011).

It is important to acknowledge that the portrayal of gender in Disney animations and media more broadly is reflected by, and a reflection of, changing attitudes toward men and women in society (Gill, 2007a). The portrayal of the princesses in situations that they wish to escape, and the influence of dominating father figures are relatively consistent themes in the “middle” princess films. Although the female protagonists tended to be strong willed and seek independence, they ultimately fall in love sometimes at the detriment of their initial dreams or goals (England et al., 2011). The notion that women will be ultimately fulfilled by their romantic unions is, for Faludi, an anti-feminist backlash message intended to refocus women's attention on marriage and the home (Faludi, 1993). Faludi's (1993) work considers the influence of repeated cycles of anti-feminist backlash on the portrayal of men and women in media that surfaces when women make gains in the fight for equality. She finds for example, after women were granted the Equal Rights Amendment in America in 1972, there was an almost instant response in which “New Right” groups argued that feminism had simply gone too far, needed to be rejected or reversed and had caused unhappiness in women, men, and families to encourage women to turn against their own cause (Faludi, 1993; Mendes, 2011). The messages regarding inevitable romance and dominating father figures in the “middle” Disney princess films may reflect the backlash Faludi identifies. Further, the masculinity of the male characters with whom the princesses in this era are romantically involved with is also consistent with backlash politics that relies on reinforcing and exaggerating the difference between men and women to justify their different social roles (Faludi, 1993; Tasker, 1993). That the “middle” Disney princess films seem to be influenced by backlash politics provides evidence for the entanglement with “innocent” Disney animations and problematic gendered messages.

So-called “modern” Disney princess animations (*The Princess and the Frog*, Clements and Musker, 2009; *Tangled*, Greno and Howard, 2010; *Brave*, Andrews et al., 2012; *Frozen*, Buck and Lee, 2013; *Moana*, Clements et al., 2016) have the most androgynous female characters, that is, those high in both feminine and masculine behaviors. These androgynous princesses tend to be highly assertive and athletic (masculine traits) as well as fearful and tentative (feminine traits; Hine et al., 2018a). The authors suggested that this movement from highly stereotypical Disney princesses to those high in both masculine and feminine behavior provides evidence that there is a wide range of female gender profiles shown in the franchise overall, and that depictions of princesses have become more progressive over time (Hine et al., 2018a). Both the “modern” and “early” princes seemed to be less stereotyped, that is, lower in masculine behavior than the “middle” princes, although it should be noted that only borderline significance was found in the change in masculine behavior displayed by princes across the three eras (Hine et al., 2018a). This suggests that the development of male protagonists' gender profiles is less linear and chronological than those of the princesses, as well as less prominent.

Postfeminist messages have been identified in Disney's portrayal of masculinity and femininity and these seem to relate most to the “modern” animations. Post-feminism often has both feminist and antifeminist sentiment as it usually finds that gender equality has largely been achieved (Gill, 2007b; Frasl, 2018). It both celebrates and overstates the accomplishment of the women's movement and assumes that its current pursuit is needless (Butler, 2013 cited by Gorton, 2004; Gill, 2007b; Frasl, 2018). Postfeminist men are often referred to as being in crisis due to conceptualizations of masculinity expanding to include feminist values (Rumens, 2017). Women, Gill (2007b) discusses, are often simultaneously sexualized in postfeminist media while presented as autonomous, in control, and often sexually liberated rather than passive. The female protagonists may be more active in postfeminist media, but may also “seem compelled to use their empowered postfeminist position to make choices that would be regarded by many feminists as problematic, located as they are in normative notions of femininity” (Gill, 2007b, p. 162). This therefore seems to present women as choosing to be traditionally feminine as autonomous subjects without investigating whether this is in fact a choice (Gill, 2007b).

In “modern” Disney animations, male protagonists are often presented as immature, inept, and the butt of the joke in contrast to their stronger female equivalents (Macaluso, 2018). Female characters in children's films seem to be more intelligent whereas men seem to be stronger and funnier (Smith et al., 2010) and men in postfeminist films are likely to be underachievers in financial trouble (Gill, 2014). These traits are particularly present within *The Princess and the Frog* (Clements and Musker, 2009) as Naveen holds many of these qualities. Other modern princes such as Flynn Rider and Kristoff also fit into this new masculine archetype (Macaluso, 2016, 2018). Although this can lead to the conclusion that Disney films with such a dynamic could be empowering because the female characters are stronger and more competent than the male characters, this is a sign of postfeminist content because:

“There seems to be a message that men must be weak in order for women to thrive. This message is dangerous to both sexes, as it subtly suggests that women and men cannot successfully coexist as strong, independent individuals together” (Macaluso, 2018, p. 8).

The implications of presenting women as strong only against weak, less masculine (Gillam and Wooden, 2008) and largely mocked male characters are unclear.

Further, by representing princesses who need the help of men to achieve their goals and maintaining a focus on female characters' appearances (best exemplified by “Let it Go” in *Frozen*, Buck and Lee, 2013), “one of the strongest features of post-feminism [is endorsed]: a contradictory articulation of progressive and regressive elements of gendered identities and identifications” (Rudloff, 2016, p. 17). Therefore, it seems that contradictory gender messages have been prevalent throughout Disney's history, but the “modern” princess films endorse postfeminist messages by portraying strong(er) female characters alongside “weak or foolish male character[s]” (Macaluso, 2018, p. 7). Highlighting the relation between the gendered messages in Disney films and the women's movement provides evidence that such messages reflect, impact upon, and/or are impacted by, the political climate in which they exist (Giroux and Pollock, 2010).

Overall, it appears that the depiction of the gendered behavior displayed by prince characters has remained largely stable over time (England et al., 2011) albeit with some evidence that the “modern” men were higher in feminine than masculine traits than their earlier counterparts. This trend warrants further research as a borderline significance was found (Hine et al., 2018a), therefore analyzing more male profiles is necessary. Disney princesses, however, seem to enjoy a more pronounced chronological progression from being highly stereotyped in the “early” animations to more androgynous (high in masculine and feminine traits) over time. Some non-princess and more recently released animations require content coding analysis to provide a more detailed appraisal of these trends. Additionally, a further appraisal of the earliest Disney princess animations is warranted as they are still heavily marketed and widely seen meaning they are likely to still be influential today. It should be noted that although there is nothing inherently wrong with females adhering to femininity (and males adhering to masculinity), and characters in mass media being portrayed to do so, androgyny for Bem and Lewis (1975) “was equated with [gender role] flexibility, and flexibility was related to adaptive and positive mental health” (Bem and Lewis, 1975, as cited by Martin et al., 2017, p. 593). Therefore, less stereotyped gender role profiles in Disney protagonists may be advantageous, especially if this is replicated by the children engaging with such media.

### 1.2 The impact of gendered messages in media

Gendered messages in mass media, such as the Disney feature length animations that have been described above, are likely to affect children's understanding of the appropriate behavior for men and women, as argued by theories such as Social Cognitive Theory of Gender Development (Bussey and Bandura, 1999) and Gender Schema Theory (Bem, 1981). Social Cognitive Theory describes the ways in which children learn about gender differentiation from their environment. The theory suggests that children observe, attend to, and replicate behavior of same-sex models more frequently because they identify them as like themselves in a process referred to as modeling. Children learn gendered information through models in their direct environment such as their parents, siblings, and peers as well as through the media. A child must be motivated to replicate behavior and motivation is likely to be lower if it is associated with an adverse outcome (for example if it is chastised) and is likely to be higher if there is a potential incentive (for example, they may get praised). Because children are more likely to be praised for behaving in accordance with their gender role stereotype, they are more motivated to replicate stereotyped behavior. Similarly, if a character in a film is shown to be displaying a gender a-typical behavior and is humiliated as a result, the child will not be motivated to display the behavior themselves. During this process, the developing child learns to “regulate their own conduct by the reactions they expected from others, pursuing same-gender activities but shunning activities linked to the other gender” (Bussey and Bandura, 1999, p. 698).

Similarly, Gender Schema theory (Bem, 1981) describes the importance of the environment in children's understanding of gender. The theory describes the process that children learn to associate information they gain into organized schemas regarding what is “for boys” and what is “for girls.” Schemas develop in complexity as more information from the environment is assimilated. The theory suggests that children are motivated to acquire knowledge to develop their gender schema when they are able to label their own gender and thus appreciate gender as a significant feature of their identity (Bussey and Bandura, 1999). While the child is assimilating information into their gender schema, they may also use this information to guide their behavior. Children gain gendered information from their environment, including the media they consume. Both Social Cognitive Theory, and Gender Schema Theory therefore suggest that the messages obtained from media will be influential to children's understanding of gender and the behavior they display.

However, although there is theoretical grounding to suggest that the gendered profiles displayed by Disney protagonists may be influential to children, there is less research that examines this phenomenon than the messages within the Disney feature length animations themselves. In a study that importantly examined whether children notice the gendered behavioral profiles of Disney princesses, Hine et al. (2018b) found that children between the ages of 8 and 9 years old rated Aurora (from *Sleeping Beauty*, Clark et al., 1959) as more feminine than Moana. This finding provided evidence that children not only observed differences in the gendered behavior of Disney protagonists, but they attributed the gendered traits to such characters in line with academic research and perspectives (Hine et al., 2018b). The participants also reported that princesses in general were more feminine than masculine (Hine et al., 2018b). This research therefore raises the question as to how children's perception that some princesses are both feminine and masculine (such as Moana), but overall, princesses are more feminine, will impact their gendered behavior.

Research investigating the impact of female Disney protagonists on children's behavior finds that there is an association between exposure to Disney princess characters and children's displays of stereotypically feminine behavior (Coyne et al., 2016; Golden and Jacoby, 2018). For example, Coyne et al. (2016) found that high levels of engagement with Disney princess media was associated with more stereotypically feminine behavior in both boys and girls and that the effect was longitudinal—it predicted the level of feminine behavior displayed after 12 months. Additionally, some researchers have provided children with Disney princess dressing-up outfits and recorded their play activities and behaviors (Golden and Jacoby, 2018) or their performance on certain tasks (Coyne et al., 2021). Although such research does not provide evidence of the association between the content in Disney films themselves and children's gendered behavior, they can provide insight into the behaviors children have learned to associate with characters from such films and thus deem appropriate to replicate when dressed like them. When provided with Disney princess outfits, girls reproduced highly feminine behavior such as focusing on their appearance and displaying highly feminized movements such as twirling (Golden and Jacoby, 2018). They also avidly excluded boys from their play which suggests that the participants marked princess play as appropriate for girls and not boys (Dinella et al., 2017; Golden and Jacoby, 2018). However, girls' toy preferences were not affected by the dressing-up outfits they wore (whether these were Disney princess outfits, superhero outfits, or gender-neutral outfits), which could reflect the more androgynous behavioral profiles of princesses in modern animations, as discussed above (Coyne et al., 2021). Conversely, boys who wore superhero outfits were less likely to show preferences for feminine typed toys than boys who wore gender-neutral outfits. Boys were also more likely to show prosocial behavior when dressed in feminine dressing-up outfits, such as that of a nurse, than when they were in superhero outfits. These findings suggest that boys were more likely to adhere to gender stereotypes when they were wearing masculine costumes than when they were wearing feminine or neutral costumes. It is interesting to note that it was revealed in pilot testing that boys often refused to wear Disney princess outfits all together, suggesting that Disney princess merchandise is strongly considered “for girls,” in line with previous work (Dinella et al., 2017).

Wohlwend (2012) also found evidence of play providing opportunities for children to challenge gender norms. When boys played as female characters in play scenarios with Disney and non-Disney dolls, their female doll characters were continuously mis-gendered. Thus, although the boys were not excluded from doll play as they were in more direct princess play (Golden and Jacoby, 2018), the more inclusionary, less gender segregated play, came with challenges for children. Wohlwend (2012) suggests that

“[t]he number of corrections in this play episode reveal how gender performances intertwined with doll play and enforced an expectation that boys could animate dolls but should not animate female characters” (Wohlwend, 2012, p. 15).

Taken together, this research suggests that Disney princess narratives are associated with more femininity in the children engaging with them (Wohlwend, 2012; Coyne et al., 2016; Golden and Jacoby, 2018) and this could explain why boys frequently refused to wear Disney princess dressing-up outfits (Coyne et al., 2021). Therefore, despite children's recognition that a more “modern” female protagonist is both feminine and masculine (Hine et al., 2018b), it is children's overall conceptualizations of princesses (Dinella et al., 2017; Hine et al., 2018b) that seem to dominate when it comes to the behavior they reproduce. More broadly, the research also implies that Disney media influences the narratives that children create in their play, as well as the behavioral profiles of both boys and girls (Wohlwend, 2012; Coyne et al., 2016). This highlights the importance of studying gendered messaging within such media. Further, much like content coding research conducted by England et al. (2011) and Hine et al. (2018a), the focus on the impact of the gendered messages within Disney animations has primarily focused on the princess franchise, and the female protagonists within that franchise. Both the impact of the prince characters and the gendered profiles of non-prince male protagonists that exist outside the princess franchise are currently not known.

### 1.3 The aims of the current study

Much of the research considering the gendered behavior displayed by Disney protagonists focuses on the Disney princess franchise. As a result, some potentially influential protagonists (for example, those from movies grossing over $200 million worldwide but not featuring a Disney princess) have not been examined with the same rigor. Perhaps particularly significantly, the previous focus on the Disney princess franchise means that many male non-prince characters have been excluded from previous research. The male protagonists that do not feature within the Disney princess animations may be among the most influential to young boys, as boys are less likely to engage with media they associate as being “for girls” (Dinella et al., 2017). Notably, Davis (2013) identified that there was an increase in leading male protagonists in the Disney animations around the 1990s (with films such as *The Hunchback of Notre Dame*, Trousdale and Wise, 1996; *Hercules*, Clements and Musker, 1997; *Tarzan*, Buck and Lima, 1999) which could reflect the company aiming to draw in a young male audience. The current study aims to examine the gendered profiles of some of these characters. Further, as new Disney animated films are released, additional content analyses are necessary to provide modernized assessments of gendered portrayals. *Frozen 2* (Buck and Lee, 2019) and *Raya and the Last Dragon* (Hall et al., 2021) were not released when Hine et al. (2018a) conducted their research. *Frozen 2* (Buck and Lee, 2019), which although a sequel, generated more profit than its prequel (McGuire, 2020; Whitten, 2020) making it worthy of research. Raya is a welcome addition to the franchise, given her Southeast Asian identity and the film's move away from the princess-meets-prince narrative (Debruge, 2021; Dzurillay, 2021; Ramella, 2021). A content coding of the leading characters in some of the most recent Disney releases, such as *Frozen 2* (Buck and Lee, 2019) and *Raya and the Last Dragon* (Hall et al., 2021) is needed.

Additionally, the content analyses conducted by England et al. (2011) and Hine et al. (2018a) also had methodological drawbacks particularly in relation to the coding scheme utilized. For example, Hine et al. (2018a) found that the *shows emotion* code was limiting. *Shows emotion* was defined as

“the expression of both positive and negative representation of feeling. This was only coded for princes because initial piloting of the coding scheme indicated princesses consistently displayed emotion at each opportunity throughout and it was unreasonable to code” (England et al., 2011, p. 599).

Although the code was adequate in coding the early Disney princess films, it was not representative of the broader emotion displayed in the later films (Hine et al., 2018a). This issue could partly reflect that the “modern” Disney men have much more screen-time than the “early” princes meaning that they are generally more developed characters (Davis, 2013). More broadly, it is possible that the progression from hand-drawn animation to the use of computer-generated imagery within the “modern” era of Disney animated feature length films allows for emotion to be expressed by characters more clearly. For example, the level of expression that is shown by the characters within *Snow White and the Seven Dwarves* (Cottrell et al., 1937) is much less pronounced than that within *Moana* (Clements et al., 2016) because the animation is much more simplistic in the former.

Further, the *shows emotion* code was applied exclusively to the behavior of the princes (and was treated as a feminine trait) which meant that emotional displays of princesses (beyond the extreme case of a princess collapsing while crying), were not coded because princesses showed emotion at high frequencies (England et al., 2011). However, because emotion is displayed at such high frequencies, coding emotion for only male protagonists could have potentially influenced the results of previous research. Further, the code did not differentiate between the emotions displayed meaning that presumably, an angry outburst could have been attributed this code as well as a scene in which a prince cried, even though the former is arguably a more traditionally masculine emotional display than the latter. The *shows emotion* code was the most frequently displayed by the prince characters in both the original (England et al., 2011) and the modern extension of the work (Hine et al., 2018a) likely reflects the variance of behavior that could fit into the code and highlights the need for specific emotional behaviors to be recorded, and in a more general sense, a review of the coding scheme applied.

In summary, pervious quantitative content analyses have provided substantial insight into the behavior of Disney prince and princess characters. However, there are three important ways to extend this research, including incorporating animations beyond the Disney princess franchise to analyze a more diverse group of protagonists, incorporating recent films not yet analyzed, and applying a revised coding framework that addresses key limitations to earlier versions.

The current study conducted a quantitative content analysis of 39 Disney protagonists spanning films released from 1937 to 2021 for their levels of stereotypically masculine, feminine and gender-neutral behavior. The study built on previous research in three ways, addressing each of the limitations referred to above. Firstly, it examined the gendered behavior displayed by a greater number of influential Disney protagonists than previous content analyses, including characters from non-princess films. Secondly, it analyzed the gendered portrayals within two of the Disney animations released since the prior work (Hine et al., 2018a) was conducted, namely *Frozen 2* (Buck and Lee, 2019) and *Raya and the Last Dragon* (Hall et al., 2021). Thirdly, the current study utilized a more expansive framework of behaviors to overcome some of the drawbacks of the one utilized by England et al. (2011) and Hine et al. (2018a). Although Hine et al. (2018a) utilized the same coding framework so that the gendered behaviors displayed by Disney characters released between 1937 and 2009 could be directly compared to those released between 2009 and 2016, this was not necessary in the current study. Therefore, the framework applied in the current study was revised by adding new behavioral codes and expanding the descriptions of others. This approach aimed to minimize some of the limitations with the previous behavioral codes. The current study was guided by the following research questions and hypotheses:

RQ1: Will both male and female protagonists be higher in feminine than masculine behavior?

H1A: Female protagonists will be higher in feminine than masculine behavior overall.

H1B: Male protagonists will be higher in feminine than masculine behavior overall.

H1C: There will be no significant difference in the masculine, feminine and gender-neutral behavior displayed by male and female protagonists when the two gender groups are compared.

RQ2: Will there be changes in the gendered profiles of male and female protagonists across the three eras analyzed?

H2: Female protagonists' gendered behavior will have changed over time. Specifically, the “modern” female protagonists will display higher levels of masculine behavior when compared to their “early” and “middle” counterparts. Conversely, the gendered behavior displayed by male protagonists will not have changed significantly over time.

## 2 Materials and methods

### 2.1 Protagonist inclusion criteria

Thirty-nine protagonists from feature length films produced by Walt Disney Animation Studios were chosen for quantitative content coding analyses of stereotypically masculine, feminine and gender-neutral behavior (see Table 1). Specifically, the central protagonists were coded from Disney animated feature length films that (a) had a central human, adult male protagonist (b) had grossed over $200 million dollars worldwide and/or (c) were associated with the Disney princess franchise. These criteria aimed to capture the most influential Disney protagonists. It should be noted here that whilst the influence of films cannot simply be measured by box-office results, worldwide grossing is arguably the most practical way of determining reach and popularity. Thus, in line with Hine et al. (2018a), the $200 million benchmark was deemed appropriate. Furthermore, children are most likely to model behavior from people or characters they deem to be like themselves (Bussey and Bandura, 1999). Therefore, Disney animated feature length films that were centered around animal protagonists were excluded, as the gendered behavioral profiles of such characters are less likely to be influential to children and are more difficult to accurately code.

**Table 1:** The films and protagonists content coded, the era to which they belong and the Kalpha reliability scores.

		**Male protagonist**		**Female protagonist**	
**Film era**	**Film name**	**Name**	**Kalpha**	**Name**	**Kalpha**
“Early”	Snow White and the Seven Dwarves (Cottrell et al., 1937)	The Prince	0.74	Snow White	0.70
	Cinderella (Geronimi et al., 1950)	Prince Charming	0.81	Cinderella	0.78
	Sleeping Beauty (Clark et al., 1959)	Prince Philip	0.79	Aurora	0.70
“Middle”	Little Mermaid (Clements and Musker, 1989)	Eric	0.68	Ariel	0.7
	Beauty and the Beast (Trousdale and Wise, 1991)	The Beast	0.73	Belle	0.73
	Aladdin (Clements and Musker, 1992)	Aladdin	0.73	Jasmine	0.7
	Pocahontas (Gabriel and Goldberg, 1995)	John Smith	0.69	Pocahontas	0.73
	Hunchback of Notre Dame (Trousdale and Wise, 1996)	**Quasimodo**	0.71	**Esmerelda**	0.71
		**Phoebus**	0.68		
	Hercules (Clements and Musker, 1997)	**Hercules**	0.73	**Meg**	0.74
	Mulan (Bancroft and Cook, 1998)	Li Shang	0.70	Mulan	0.72
Tarzan (Buck and Lima, 1999)	**Tarzan**	0.70	**Jane**	0.84
“Modern”	The Princess and the Frog (Clements and Musker, 2009)	Naveen	0.74	Tiana	0.73
	Tangled (Greno and Howard, 2010)	Flynn	0.75	Rapunzel	0.73
	Frozen (Buck and Lee, 2013)	Kristoff	0.77	Elsa	0.77
		Hans	0.74	Anna	0.77
	Moana (Clements et al., 2016)	**Maui**	0.77	Moana	0.72
	Frozen 2 (Buck and Lee, 2019)	**Kristoff**	0.73	**Elsa**	0.78
			**Anna**	0.79
Raya and the Last Dragon (Hall et al., 2021)	**Father Benja**	0.78	**Raya**	0.8
			**Numaari**	0.69

### 2.2 The coding framework

The coding framework utilized in the current study consisted of 52 codes and England's et al. (2011) previously established framework (later used by Hine et al., 2018a) was evaluated and adapted. The full coding framework is available as Supplementary material. This section will describe and justify the adaptations made to the previous behavioral codes and discuss codes that were added for the current study.

Four behavioral code descriptions (*athletic, nurturing, collapses crying*, and *ashamed/guilty*) remained the same in the current study as they appeared in the previous study. These code descriptions were clear and seemed applicable to all eras of animation and thus did not require adaptation. Although the description of *ashamed* from previous studies remained unchanged in the current work, the code name was changed from (ashamed to *ashamed/guilty*) to represent the description more accurately.

Six behavioral codes were created by dividing previous code descriptions into more than one behavior in the current framework. Some of these represented nuanced differences in a behavior that could change its meaning. For example, England et al. (2011) stated that although princesses expressed assertiveness frequently, they were rarely assertive to other adult protagonists and this arguably portrayed “a fairly submissive and limited way of being assertive” (England et al., 2011, p. 562). Therefore, separate *assertive toward adults* and *assertive toward children/animals* behavioral codes were incorporated into the current framework, the former as a masculine code and the latter as a feminine one.

Eighteen behavioral codes that featured in England et al. (2011) and Hine et al. (2018a) underwent relatively minor changes, mainly to ensure that the descriptions were more explicit about how a behavior may appear within a target film, and in some cases, to make the behavioral code broader. Also, some adjustments to pre-existing codes reflected the need to ensure that the descriptions did not overlap due to the higher number of codes incorporated into the current framework (52 as opposed to the previous 27). An example of a minor adjustment includes the addition of climbing to the *physically strong* code description. Some behavioral codes underwent more major adjustments for the purpose of the current study with their descriptions being partially or completely re-written. For example, the first author felt that the previous *curious about the princess* code that had the description “exhibiting a studious, concerned expression when looking at the princess. This behavior suggested that the female had a mystique that was captivating and romantically compelling” (England et al., 2011, p. 558–559) could be subjective and felt that it was unclear what could constitute a “studious, concerned expression.” Therefore, the code name was changed to *shows romantic interest* and the description was re-written. The new description included examples such as, a character deliberately engaging in a conversation with the intention of getting to know another character in a way that suggested romantic interest, as well as a character being unable to control themselves around a love interest (appearing glazed over or looking mesmerized by them). These changes were informed by notes that were taken on the gendered messages within Disney animated feature length films before data collection for this study had begun.

Twenty-three codes that did not appear in the previous framework were added to the current framework. The number of new behavioral codes in current study reflects the aim to code as much of the behavior displayed by the protagonists as possible so that it could comment upon more complete gender profiles. The new codes included six emotion-based codes such as *expresses positive emotions (excluding excitement), sad, angry/frustrated*, and *panic*, which were added due to the drawback of the *shows emotion* code in the previous framework (Hine et al., 2018a) as discussed in the previous section of this paper. Other new codes included *deceitful, honest*, and *charming*. These had not been included in previous work conducted by England et al. (2011) and Hine et al. (2018a) but were identified utilizing a bottom-up approach during which Disney (target and non-target) films were viewed before the study had been designed and behaviors that were exhibited frequently across multiple films were noted. To generate the new codes and create accurate code descriptions, two of the authors had several meetings where behavior that occurred in several Disney animated films but was not captured in the previous coding framework was identified and discussed. During the meetings, scenes in which the behavior that did not fit within the previous coding framework were rewatched to ensure that descriptions and code names generated would represent the behavior as it appeared in the relevant text and that the examples provided would facilitate the application of that behavioral code. When all the changes had been made to the coding framework it was approved by a third author.

Two codes that were present in the previous framework were removed and these decisions were also made via discussions and meetings between the authors, as described above. The *gives advice* code was incorporated into the description of the *helpful* code. One (rather than two) *described as physically attractive* code was included in the current study and was considered a feminine trait. It was not deemed necessary to have separate described as attractive codes for male and female protagonists as the principal of being described as attractive is arguably the same regardless of gender. Further, previous research finds that female Disney characters' appearances are given more value than males' (Towbin et al., 2004). It therefore seemed appropriate to deem being described as physically attractive a feminine trait.

The gendered split of the behaviors in the coding scheme was based on previous research (Thompson and Zerbinos, 1995; Do Rozario, 2004; England et al., 2011). To further our understanding of the behavioral profiles of Disney protagonists, the current study considered the portrayal of gender-neutral behavior in addition to the commonly studied gender typical and atypical behavior. The frequency of gender-neutral behavior has not been explicitly examined. It was important to include such behavior in the current analysis to establish whether most of the behavior displayed by Disney protagonists was indeed gendered.

### 2.3 The coding procedure

All the films were coded by at least two coders (with the majority, 13 of 17, being coded by three coders). Two lead coders (referred to as the primary and second coders throughout this section) coded all the target protagonists. The lead coders were trained utilizing two films that were excluded from the study but represented similar content for coding (i.e., *Brave*, Andrews et al., 2012 and *Atlantis: The Lost Empire*, Trousdale and Wise, 2001). Three additional coders also coded *Atlantis: The Lost Empire* (Trousdale and Wise, 2001) for training purposes and coded between 8 and 10 target protagonists each. The target films that were coded by three coders were reviewed in two stages. First, the opening 15 minutes of each of the target films was coded and discrepancies were discussed until Krippendorf's alpha (hereafter, Kalpha) reached 0.67 (Hayes and Krippendorff, 2007). The remainder of the film was then coded, and Kalpha's were calculated for the full behavioral profile of each character, and if necessary, further discussions were had to improve the inter-rater reliability score.

The primary coder acted as the reviewer of the coding in which discrepancies were highlighted. If an agreement between coders could be reached, the coding was amended, but if the coders fundamentally disagreed on the interpretation of a behavior, the coding was left as it was. This approach ensured that reliability was maintained for each protagonist while honoring each coder's interpretation. It is possible that having the primary coder conducting those discussions could have created a bias in that they may have been more likely to perceive their own coding as accurate. However, the discussions were deliberately collaborative. It is also possible that the primary coder leading discussions facilitated more critically aware coding procedures and ensured that the researcher was close to, and engaged with, the methodological approach that was followed at all stages of the research.

The films coded by the two lead coders were coded in their entirety, and the opening 15 minutes of the coding was not reviewed first, due to their attendance of extra training sessions. To monitor the reliability throughout the process of data collection, as more results were added into the analyses, reliability was calculated and reported for each protagonists' behavioral profile (see Table 1 for final Kalpha values).

The coding procedure replicated that previously established by England et al. (2011) and Hine et al. (2018a). A protagonist was attributed a code when they were seen to display a trait/behavior in the framework, or they were mentioned to possess a trait. The same behavior was coded again if the scene changed, and the behavior was still being displayed. However, if the same behavior was displayed more than once within one scene, it was only coded once. Multiple codes could be attributed to a behavior. For example, if a character was angry while vocalizing that they were not interested in another character romantically, both an *angry/frustrated* and an *uninterested in love* code could be recorded to accurately capture the behavior displayed in that moment. Each code was timestamped and tallied meaning that a frequency could be calculated for each behavioral code when the coding of each protagonist was completed.

## 3 Results

Before any analyses were conducted, the total number of the masculine, feminine, and gender-neutral behaviors counted by each coder for each protagonist were averaged. The averages for these three sets of behaviors were then converted into percentages of each protagonists' total number of behaviors to mitigate for variance in the total frequency of behavioral codes attributed for each protagonist. Such an approach was previously utilized by England et al. (2011) and Hine et al. (2018a) and is particularly important when comparing “early” protagonists to “middle” and “modern” protagonists who tended to have more codable behavior (due to changes in animation style). The percentages of masculine, feminine, and gender-neutral behaviors were then entered into SPSS, and the percentages of the gendered behavior displayed by each protagonist across each of the coded films can be found in Table 2. The categorization of the films into these eras is consistent with England et al. (2011) and Hine et al. (2018a).

**Table 2:** The percentages of gendered behavior displayed by male and female protagonists.

	**Male protagonist's behavior**				**Female protagonist's behavior**			
**Film name**		**Masculine %**	**Feminine %**	**Neutral %**		**Masculine %**	**Feminine %**	**Neutral %**
Snow White and the Seven Dwarves (Cottrell et al., 1937)	The Prince	47.83	52.17	0	Snow White	16.99	76.74	6.27
Cinderella (Geronimi et al., 1950)	Prince Charming	22.03	72.88	5.09	Cinderella	16.31	72.81	10.87
Sleeping Beauty (Clark et al., 1959)	Prince Philip	44.92	47.46	7.63	Aurora	9.38	79.89	10.72
The Little Mermaid (Clements and Musker, 1989)	Eric	27.56	57.72	14.72	Ariel	19.28	70.58	10.14
Beauty and the Beast (Trousdale and Wise, 1991)	The Beast	44.49	47.27	8.24	Belle	34.46	53.68	11.87
Aladdin (Clements and Musker, 1992)	Aladdin	29.46	56.53	14.01	Jasmine	37.56	48.08	14.36
Pocahontas (Gabriel and Goldberg, 1995)	John Smith	37.61	53.98	8.39	Pocahontas	33.42	56.80	9.78
The Hunchback of Notre Dame (Trousdale and Wise, 1996)	**Quasimodo**	29.07	61.23	9.70	**Esmerelda**	33.04	59.50	7.46
	**Phoebus**	37.30	53.99	8.71				
Hercules (Clements and Musker, 1997)	**Hercules**	39.36	48.69	11.94	**Meg**	39.61	48.69	11.94
Mulan (Bancroft and Cook, 1998)	Li Shang	58.77	28.83	12.39	Mulan	27.49	56.90	15.60
Tarzan (Buck and Lima, 1999)	**Tarzan**	43.31	48.00	8.69	**Jane**	25.78	66.06	8.17
The Princess and the Frog (Clements and Musker, 2009)	Naveen	28.35	60.78	10.87	Tiana	31.96	54.12	13.91
Tangled (Greno and Howard, 2010)	Flynn	29.77	52.19	18.04	Rapunzel	28.30	60.09	11.61
Frozen (Buck and Lee, 2013)	Kristoff	31.66	56.68	11.66	Anna	27.32	62.42	10.26
	Hans	30.44	51.06	18.50	Elsa	35.71	56.39	7.90
Moana (Clements et al., 2016)	**Maui**	50.93	35.98	13.10	Moana	31.42	51.46	17.13
Frozen 2 (Buck and Lee, 2019)	**Kristoff**	22.57	60.62	16.81	**Anna**	25.77	60.69	12.65
					**Elsa**	34.47	55.59	12.65
Raya and the last Dragon (Hall et al., 2021)	**Father**	31.76	55.59	12.65	**Raya**	30.85	55.03	14.12
	**Benja**				**Numaari**	45.89	40.98	13.13

### 3.1 Comparing the gendered profiles of male and female protagonists

To answer hypotheses 1A and 1B, paired sample *t*-tests were conducted to reveal whether male and female protagonists were significantly higher in either masculine or feminine behavior within their own character sex. Table 3 shows the mean scores for male and female protagonists on masculine, feminine, and gender-neutral behavior. Results revealed that both male, *t*_(18)_ = 3.80, *p* < 0.01, and female protagonists, *t*_(19)_ = 7.361, *p* < 0.001, were significantly higher in feminine behavior than masculine behavior overall. According to the means, 36.17% of male protagonists' behavior was masculine and 52.72% was feminine. 29.25% of females' behavior was masculine and 59.38% was feminine. Both Hypothesis 1A and 1B were therefore supported.

**Table 3:** The mean percentages of gendered behavior displayed by male and female protagonists.

**Gendered behavior**	**Female protagonists**	**Male protagonists**
Masculine	29.25	36.17
Feminine	59.38	52.72
Gender-neutral	11.37	11.11

In answer to hypothesis 1C an independent *t*-test comparing the percentage of masculine behavior displayed by male and female protagonists revealed that, contrary to the prediction made, male protagonists were significantly higher in masculine traits (M = 36.17%) than female protagonists (*M* = 29.25%), *t*_(37)_ = 2.306, *p* < 0.05. A second independent *t*-test comparing the percentage of feminine behavior displayed by male and female protagonists revealed that there was also a significant difference between male and females, *t*_(37)_ = 2.157, *p* < 0.05 with male protagonists being significantly lower in feminine traits (M = 52.71%) than females (M = 59.38%).

Although no hypotheses were made regarding the percentage of gender-neutral behavior displayed by male and female protagonists because of a lack of research on the topic, analysis was also conducted on these results. Because both male and female characters were shown to be more feminine than masculine, the percentage of gender-neutral behavior was compared to the masculine percentages for both male and female characters. Assessing this would determine whether masculine behavioral portrayals also differed significantly from the portrayal of gender-neutral behavior which, according to the means, were displayed the least by both male and female protagonists. It was revealed that both male *t*_(18)_ = 8.887, *p* < 0.001 and female protagonists *t*_(19)_ = 9.431, *p* < 0.001 showed higher percentages of masculine than gender-neutral behavior, as gender-neutral behavior accounted for 11.11% of male protagonists' gender profiles, and 11.37% of females'. This confirmed that gender-neutral behavior was the least portrayed for both male and female protagonists, providing evidence that most of the behavior displayed by Disney protagonists is indeed gendered. This reaffirms the need for studying the gendered behaviors displayed by such protagonists.

### 3.2 Changes over time

The current study also aimed to examine whether Disney protagonists' gendered behavior had changed over time. To assess this, One-Way Analysis of Variances (ANOVAs) were conducted. For each, the Disney era was used as the independent variable (with three levels “early,” “middle,” and “modern”). Dependent variables were the percentage of masculine behavior displayed by the female protagonists in the first ANOVA, the percentage of feminine behavior for the second, and the percentage of neutral behavior displayed by female protagonists in the third. The tests were then conducted in the same way using the percentages of masculine, feminine and neutral behavior for the male protagonists. Table 4 shows the mean percentages of masculine, feminine and gender-neutral behavior displayed by the protagonists in each era. For additional information, Table 5 displays the total number of behaviors coded for each protagonist.

**Table 4:** The mean percentages of gendered behavior displayed by male and female protagonists in each era.

	**Female protagonists**			**Male protagonists**		
**Era**	**Feminine**	**Masculine**	**Gender-neutral**	**Feminine**	**Masculine**	**Gender-neutral**
Early	76.48	14.23	9.29	57.50	38.26	4.24
Middle	57.65	31.33	11.02	50.69	38.55	10.75
Modern	55.23	32.41	12.36	53.27	32.21	14.52

**Table 5:** The numbers of behaviors coded for each protagonist.

	**Male protagonist**		**Female protagonist**	
**Film name**	**Name**	**Number of behaviors**	**Name**	**Number of behaviors**
Snow White and the Seven Dwarves (Cottrell et al., 1937)	The Prince	23	Snow White	239
Cinderella (Geronimi et al., 1950)	Prince Charming	39	Cinderella	282
Sleeping Beauty (Clark et al., 1959)	Prince Philip	118	Aurora	124
Little Mermaid (Clements and Musker, 1989)	Eric	231	Ariel	463
Beauty and the Beast (Trousdale and Wise, 1991)	The Beast	324	Belle	553
Aladdin (Clements and Musker, 1992)	Aladdin	693	Jasmine	390
Pocahontas (Gabriel and Goldberg, 1995)	John Smith	358	Pocahontas	385
The Hunchback of Notre Dame (Trousdale and Wise, 1996)	Phoebus	272	**Esmerelda**	384
	Quasimodo:	615		
Hercules (Clements and Musker, 1997)	**Hercules**	943	**Meg**	395
Mulan (Bancroft and Cook, 1998)	Li Shang	272	Mulan	673
Tarzan (Buck and Lima, 1999)	**Tarzan**	633	**Jane**	363
The Princess and the Frog (Clements and Musker, 2009)	Naveen	770	Tiana	793
Tangled (Greno and Howard, 2010)	Flynn	693	Rapunzel	727
Frozen (Buck and Lee, 2013)	Kristoff	498	Anna	912
	Hans	260	Elsa	462
Moana (Clements et al., 2016)	Maui	756	Mona	1222
Frozen 2 (Buck and Lee, 2019)	Kristoff	339	Elsa	707
			Anna	683
Raya and the Last Dragon (Hall et al., 2021)	Father Benja	170	Raya	861
			Numaari	347

Hypothesis 2 was only partially upheld for female protagonists as the first ANOVA revealed that the percentage of masculine behavior was significantly different across the three eras, *F*_(2, 17)_ = 10.679, *p* < 0.001, as predicted. However, Tukey *post-hoc* test revealed that the “middle” (M = 31.33%) and “modern” (M = 32.41%) female protagonists did not significantly differ in their levels of masculine behavior (*p* > 0.05). The “early” female protagonists (M = 14.23%) were however significantly less masculine than the “middle” (*p* < 0.01) and “modern” females (*p* < 0.001).

A second ANOVA revealed that the percentage of feminine behavior displayed by female protagonists also differed across the three eras *F*_(2, 17)_ = 11.723, *p* < 0.001. The results of Tukey *post-hoc* tests for feminine behavior across the eras mirrored those reported for the masculine behavior in that the “middle” (M = 57.65%) and “modern” (M = 55.23%) females were not significantly different in their portrayal of feminine behaviors (*p* > 0.05), whereas the “early” females were significantly more feminine (M = 76.48%) than both the “middle” (*p* < 0.01) and “modern” females (*p* < 0.001). Paired *t*-tests comparing the percentage of masculine and feminine behavior displayed by female protagonists at each time point were conducted to examine whether females were more feminine than masculine at each era studied, as the means suggested. The results showed that the “early” *t*_(2)_ = 14.705, *p* < 0.01, “middle” *t*_(7)_ = −5.255, *p* < 0.01, and “modern” females *t*_(8)_ = 5.678, *p* < 0.001 were all higher in feminine than masculine traits. Although no hypotheses were made for changes in gender-neutral behavior over time, a third ANOVA revealed that there was no significant difference in the percentage of gender-neutral behavior displayed by female protagonists across each era, *F*_(2, 17)_ = 1.463, *p* > 0.05.

The first ANOVA conducted for male protagonists revealed that the percentage of masculine behavior they displayed was not significantly different across the three eras, *F*_(2, 16)_ = 0.852, *p* > 0.05 despite an inspection of the means suggesting that the “early” (M = 38.26%) and the “middle” (M = 38.55%) male protagonists were more masculine than the “modern” males (M = 32.21%). Similarly, there was no significant difference in the percentage of feminine behavior displayed by male protagonists across the three eras, *F*_(2, 16)_ = 0.569, *p* > 0.05. These results taken together support the prediction made in hypothesis 2 regarding male protagonists' gendered behavior remaining stable across the three eras. Again, although no predictions were made for changes in gender-neutral behavior for male protagonists over time, an ANOVA revealed that the percentage of gender-neutral behavior was significantly different across the three eras, *F*_(2, 16)_ = 12.547, *p* < 0.001. Tukey *post-hoc* tests revealed the “early” male protagonists were lower in gender-neutral behavior (M = 4.24%) than the “middle” (M = 10.76%, *p* < 0.05) and “modern” male protagonists (M = 14.51%, *p* < 0.001). The “middle” and “modern” protagonists were not significantly different (*p* > 0.05).

Paired *t*-tests comparing the percentage of masculine and feminine behavior displayed by male Disney protagonists at each time point were also conducted. These revealed that the “early” male protagonists were not significantly different in their displays of masculine and feminine behavior, *t*_(2)_ = 1.217, *p* > 0.05. The “middle” male protagonists were not significantly different in their displays of masculine and feminine behavior, *t*_(8)_ = −1.911, *p* > 0.05 when the two tailed significance is reported but were significantly more feminine than masculine if their one-tailed significance was to be reported. The “modern” male protagonists were higher in feminine than masculine behaviors *t*_(6)_ = 3.272, *p* < 0.05.

## 4 Discussion

The current study investigated the gendered behavior displayed by central human adult male and female protagonists in influential Disney animated feature length films. It aimed to address three limitations of previous content analyses conducted by England et al. (2011) and Hine et al. (2018a). Firstly, it analyzed the gendered behavioral profiles of some influential protagonists that had been excluded from the previous studies. Secondly, it included protagonists from some Disney animated films that have since been released. Thirdly, it analyzed the protagonists' behavioral profiles with an updated and expanded framework, which included some gender-neutral behaviors. By including some gender-neutral behaviors in the framework, more complete behavioral profiles were captured. As a result, the current study arguably provides the most complete quantitative content analysis of Disney protagonists to date.

### 4.1 The portrayal of female Disney protagonists

The results of the current study suggest that female Disney protagonists are consistently portrayed stereotypically. For example, the female protagonists were portrayed as higher in feminine than masculine behavior overall and within each timepoint. However, there was a noticeable shift in the portrayal of female protagonists between the “early” era, where they were very high in feminine behavior, and the “middle” era, where they became less feminine and more masculine, and this has been maintained into the “modern” era. Contrary to Hine et al. (2018a), “modern” female protagonists in the current work were still more feminine than masculine overall suggesting continued stereotyping. Hine et al. (2018a) found evidence for more balanced and androgynous behavioral profiles (i.e., those high in both masculine and feminine behaviors) in female protagonists in their research. This discrepancy likely reflects the updated coding scheme applied in the current research, particularly in relation to the newly added emotion-based codes. Emotion had not been coded by England et al. (2011) or Hine et al. (2018a) for princess characters and this was a significant proportion of the behavior displayed by female protagonists in this work. Many of the emotion codes were deemed feminine, as informed by previous research (Thompson and Zerbinos, 1995; Do Rozario, 2004). This therefore provides evidence for the importance of including such codes for both male and female characters.

Although the “middle” and “modern” female protagonists did not significantly differ from each other in their displays of masculine and feminine traits, there are some noticeable differences in the narratives associated with the latter, when compared to their earlier counterparts that are not necessarily captured in the results of the current framework. Perhaps most noticeably, two “modern” films have no romantic storylines whatsoever (*Moana* Clements et al., 2016 and *Raya and the Last Dragon* Hall et al., 2021), whereas romance is key to the other films that were included in this study (Martin and Kazyak, 2009). Indeed, this has attracted media attention (such as Ramella, 2021) which suggests this warrants discussion.

Numaari, Raya (both from *Raya and the Last Dragon*, Hall et al., 2021), Moana (from the film with the same name), and Elsa (from *Frozen*, Buck and Lee, 2013 and *Frozen 2* Buck and Lee, 2019) are all “modern” female protagonists that displayed no romantically focused behaviors in the current study (coded as *wants to find romantic love* or *shows romantic interest*). For Ramella (2021), that some of the “modern” princesses are more focused on self-discovery and serving their communities than they are on pursing romantic relationships means that “they represent a more accurate reflection of modern girls and women. These princesses teach young girls to be strong and independent on their own” (Ramella, 2021, p. x). The plot of *Raya and the Last Dragon* (Hall et al., 2021) focuses on complicated female relationships, and ultimately, it is the two leading female protagonists' friendship that enables peace to be restored throughout Kumandra, something that is rarely depicted in Disney animations or in films more broadly (Radulovic, 2021). Additionally, Anna is lower in the romantically focused codes in *Frozen 2* (Buck and Lee, 2019) than she was in *Frozen* (Buck and Lee, 2013) whereas the opposite is true for Kristoff, suggesting that the portrayal of romance has changed from the prequel to the sequel. Much of the romantic plot in the latter is based around Kristoff agonizing about the best way to propose to Anna, as well as him feeling insecure that they are growing apart (as Anna embarks on a mission to release the Northoldra people from the forest in which they are trapped and leaves him behind). The fact that romance seems to be becoming less prominent in “modern” female protagonists' behavioral profiles could be a positive sign, especially for young girls who are likely to be influenced by them (Coyne et al., 2016; Golden and Jacoby, 2018).

However, although some of the “modern” animations seem to be moving away from portraying female protagonists who are motivated by their romantic pursuits, the representation of such characters could still be a reason for concern. For example, *Frozen* (Buck and Lee, 2013) has been criticized for portraying Elsa as both a leader of her people and single romantically (Streiff and Dundes, 2017a). No male character expresses romantic interest in Elsa in either of the Frozen films, which could portray that women are simply unable to occupy powerful leadership positions whilst having successful romantic relationships, which is exemplified in Anna and Kristoff's romantic plot running alongside Elsa's non-romantic one (Streiff and Dundes, 2017a). Similarly, in *Raya and the Last Dragon* (Hall et al., 2021), Numaari is frequently shown as a leader, and Raya is determined to save her people who have been turned to stone, thus the message that Disney women who are powerful and/or shown to pursue adventures cannot also have romantic relationships (Streiff and Dundes, 2017a) seems to be supported in the most recent release analyzed. Although Anna may get close to challenging this norm as she takes over from Elsa as ruler of Arendelle and is engaged to Kristoff at the end of *Frozen 2* (Buck and Lee, 2019), her leadership role given to her by her more powerful sibling which arguably reduces its significance. Therefore, the

“modern [Disney] heroine still follows certain rules that do not subvert male dominance: that is, her independence from men means that she should not threaten a man's status as the metaphorical person in the driver's seat of a relationship” (Dundes et al., 2018, p. 22).

Arguably, although Disney seem to be moving away from presenting female protagonists whose sole focus is romance, there are some problematic elements to this, as powerful female characters have yet to be portrayed as romantically involved with a partner also, which aligns with postfeminist themes (Streiff and Dundes, 2017a). Therefore, although there was no significant difference in the portrayal of gendered behavior between the “middle” and “modern” female protagonists the depiction of the two groups of female characters has some unique limitations.

It should also be noted that although the current study did not explicitly focus on language use in the Disney animations analyzed, researchers have suggested that there is stereotyping presented in the way that Disney characters converse and the dialect they speak. For example, Lippi-Green (2012) notes that female protagonists who are labeled “lovers and mothers” tend to speak “Standard American English” suggesting that characters with more diverse accents are not associated with the “goodness” necessary to occupy these highly feminized roles. Conversely, there are some (although still a small minority) of male characters who are deemed “potential lovers” that speak with “working-class” accents. It is thus important to acknowledge that the stereotyping of Disney characters is often intersectional and may be even more layered than the current results capture.

### 4.2 The portrayal of male Disney protagonists

The results of the current study found that male protagonists' behavior was more feminine than masculine overall, contrary to gendered stereotypes. Specifically, the male protagonists from the “modern” and “middle” eras were significantly more feminine than they were masculine (when the one-tailed significance is reported). Further, no significant difference was found in the displays of masculine or feminine behavior across the time points suggesting that the portrayal of Disney men has remained relatively stable. This finding partially supports the results obtained by Hine et al. (2018a), where, although it appeared that the “modern” and “early” princes were lower in masculine behavior than the “middle” princes, only marginal significance was found. Further, England et al. (2011) found no significant difference in the portrayal of gender in male protagonists across the three eras, in line with the results of the current study. Additionally, the “modern” Disney men analyzed in the current study were higher in gender-neutral behavior than the men in the other eras which provides further evidence that such protagonists may be less limited by gender stereotypes. The representation of feminine male protagonists in Disney animations may be positive as masculinity is related to aggression in children (Fehr and Russ, 2013, as cited in Coyne et al., 2014) and may be associated with poor mental health and higher suicide rates in adult men (Swami et al., 2008).

By portraying male protagonists as more feminine than masculine overall, Disney may be suggesting that men can and should deviate from their prescribed gender roles. Alternatively, it is possible that Disney is only delivering the presentation of feminine traits when male protagonists display more narratively powerful masculine traits to “compensate.” Moreover, it is unclear from the current study whether the feminine behaviors that are portrayed are received positively or negatively by other characters (and indeed viewers). If feminine men are mocked and belittled (as suggested by Macaluso, 2018) it is likely that such portrayals are still ultimately encouraging a more stereotyped (i.e., masculine) behavioral profile in young boys (Hine et al., 2018a) through the chastisement of feminine expressions. Finally, it is also unclear how the behavior of male protagonists helps shape female viewers perceptions of men and their accepted behavior, and whether they are influenced (if at all) in their perceptions of acceptable male attributes. It is therefore clear that male protagonists' feminine and masculine behaviors warrant further discussion.

Although not directly studied in this research in a quantitative manner, it is possible to consider the behaviors that may be the most celebrated in Disney men. It seems that particularly in films such as *Hercules* (Clements and Musker, 1997), male protagonists' masculine behavior is the most celebrated. For example, within the film, Hercules is adored by his fans for his physical strength and heroism (masculine traits) that he spends a significant amount of time in the film striving to achieve after being mocked and excluded for being clumsy and unintentionally troublesome (feminine traits). This is highlighted when a large statue and toy figurines are revealed of him and in both, he is presented in a posed position with his biceps flexed, exaggerating his physique, a trait that leads many of his (female) fans within the film to adore him. Further, he is motivated to develop these masculine traits in the hope that he will earn a place among the Gods, a substantial position of power (such powerful positions are also associated with masculinity). However, at the end of the film he sacrifices his opportunity to join the Gods to remain with Meg and thus chooses his romantic relationship over a position of power, which is, arguably, unlike other male Disney protagonists (Primo, 2018). It is possible that the film was less successful than anticipated because by sacrificing his masculine characteristics and goals, Hercules' character was less appealing to audiences (Primo, 2018). It seems then that Hercules' masculine traits are portrayed as the most admirable in the film.

Further, Quasimodo saves Esmerelda at the end of *The Hunchback of Notre Dame* (Trousdale and Wise, 1996); yet importantly, he must then be saved from falling to his death by the more masculine protagonist, Phoebus, who is also able-bodied). In the final scene of the film Quasimodo is embraced and carried off by the people of Paris as a sign of their acceptance of him which may not have been the case had he not shown masculine attributes such as bravery and physical strength during the scene in which he rescues Esmerelda. Arguably, such narratives suggest that although Disney men may be more feminine than masculine, they are ultimately accepted by their peers, and arguably, the audiences, only when they prove that they can also conform to the expectations of their gender role. It could also be the case that Disney men are only able to portray feminine traits when they also portray masculine ones, such as bravery and strength.

Conversely, some Disney men seem to be rewarded for their stereotypically feminine behavior. For example, Aladdin believes that by granting the Genie his freedom he is sacrificing being with the woman he loves (which has been his sole motivation throughout the entire film). He is rewarded soon after when his soon-to-be-father-in-law changes the law to make his marriage to Jasmine possible. Thus, unlike Hercules, Aladdin's selfless sacrifice (more aligned with femininity than masculinity) does not prevent him from achieving his dreams. Alternatively, Naveen and Flynn are both initially motivated by money and success, both rather masculine pursuits, however, in both films, these dreams are ridiculed. For example, in *Tangled* (Greno and Howard, 2010) Flynn's dream of making money and living in a castle is overtly scorned in an iconic scene where other physically hypermasculine men share their more effeminate dreams (such as of becoming interior designers and pianists), making the case that men with more feminine pursuits may be celebrated within the film. Both Flynn and Naveen realize throughout their respective films that love is more important than money and by doing so, Flynn marries Rapunzel, ironically becoming a prince and presumably achieving his dream in the process, despite it being previously ridiculed. Naveen, however, is shown to work in Tiana's restaurant at the end of *The Princess and the Frog* (Clements and Musker, 2009), a less glamorous end to his story. Thus, it seems that in Disney films, men shifting their focus from masculine to feminine endeavors is only sometimes rewarded. As a result, it is unclear as to whether young boys identifying with these male protagonists will deem their femininity as worthy of replication. Overall, future and perhaps qualitative research should examine the value given to male Disney protagonists' masculine and feminine behavior so that the significance of them being higher in feminine traits as a group, can be more fully understood.

### 4.3 Implications

Considering that Disney princess animations are seen to influence the gendered behavior displayed by children (Coyne et al., 2016; Golden and Jacoby, 2018), it is likely that the gendered messages within the films considered in the current study will also be impactful on children. Parents and educators could perhaps be trained in how to utilize Disney media to minimize their negative influence and maximize their potential benefits, and to become more critically aware of the messages within this media. More specifically, although a greater investigation as to whether feminine males are celebrated within Disney films is needed, that the films represent male characters who are feminine (particularly within the “middle” and “modern” animations), means they could be used to facilitate important conversations with children. For example, because some of the most frequently displayed feminine behavior by Quasimodo in *The Hunchback of Notre Dame* (Trousdale and Wise, 1996) included showing affection, displaying positive emotion, and expressing sadness, there is tentative evidence to suggest that the film could be used as an educational tool to discuss the benefits of boys and men displaying such behaviors with children.

There is also an implication provided by the results in this paper that our discourse on the presentation of gender in Disney movies may in fact constitute some form of “moral panic” (Cohen, 2011). Indeed, whilst statistically different in many cases, overall, the “gaps” in masculine and feminine presentation were not as large as in previous studies on the same movies, due to a more robust coding framework. It could be the case that the interpretation of the results is focused too heavily on difference rather than similarity between characters' behavioral profiles However, it should also be noted that many of these messages are translated to consumers in ways not measured in this study (tone, intonation, etc.) so the potential dismissal of concern should be taken with caution.

### 4.4 Limitations

The current study had several limitations. Firstly, the inclusion criteria for the current study utilized the benchmark of films that had grossed over $200 million worldwide which mirrored that of Hine et al. (2018a). However, it is important to note that this figure did not account for the popularity of Disney films on streaming services, which is a limitation. Including the number of views Disney films have had on streaming sites may have made the popularity estimates more accurate and reflected more modern viewing habits. However, streaming services have different platforms in different regions. Therefore, it would be a challenge to assess the success of a film using data from streaming services, as the number of views would differ based on the researcher's location. Therefore, although utilizing box-office grossing figures may have been a limitation, it does arguably represent the worldwide success and popularity of a film, rather than its success in one region (for example, on the UK version of Disney+). That being said, future research should perhaps work to create a new inclusion criterion that considers a combination of box-office figures and streaming success.

A discussive approach to reliability was adopted in the current study, which could be perceived as a limitation. However, the discussive approach taken was advised by members of the research team who were experienced in content coding research. Although having a more objective approach to reliability analysis may have been advantageous (Krippendorff, 2018), arguably, when coders undergo training in content analysis research, objectivity is already lost as discussions are had during those sessions (Neuendorf, 2011). Arguably then, true objectivity is not possible in content analysis studies, and a discussive approach can lead to more insightful coding in which nuanced behaviors may be more accurately captured.

Additionally, reliability was calculated for each character rather than for each behavioral code, which could be a limitation as this differed from the approach taken by Hine et al. (2018a). However, it was important for the researcher to monitor the inter-rater reliability as the coding was being conducted. Because the coding was being conducted and reviewed one protagonist at a time in the current study, the reliability of the individual behavioral codes would change as more protagonists were coded and added to analysis. Therefore, conducting reliability analysis in this way ensured that each protagonist was coded reliably.

The data collected in the current study does not consider whether stereotypical or non-stereotypical behavior displayed by Disney protagonists is presented as socially desirable or appropriate and thus worthy of replication by children who identify with them. The extent to which non-stereotypical vs. stereotypical behaviors are celebrated within Disney animations could provide further insight into Disney's motivation in presenting consistently feminine female *and* male protagonists. Perhaps this could be addressed in future research whereby the framework of behaviors established in this study could be split into “positive” and “negative” traits as well as feminine, masculine and gender-neutral. Alternatively, qualitative research could be conducted to interpret how gendered behavior is presented.

Further, although this study included male and female protagonists that had been excluded in the content analyses conducted by England et al. (2011) and Hine et al. (2018a) there has been no research examining the extent to which such Disney protagonists (i.e., those beyond the princess franchise) influence children's conceptualization of gender, or their gendered behaviors. Therefore, future research investigating the potential relationship between engagement with non-princess Disney animations and children's behavior is warranted. Perhaps a replication of Hine et al. (2018b), examining whether children recognize the feminine traits displayed by male protagonists would be a good place to start.

## 5 Conclusions

This research suggested that female protagonists throughout each era of Disney films studied are portrayed stereotypically i.e., highest in feminine traits. This finding is arguably troubling, and such results counter the perhaps more optimistic ideology posited by Hine et al. (2018a) that Disney is portraying less limited and stereotyped female characters in their “modern” animations (although they support much qualitative research such as Streiff and Dundes, 2017a,b). Although there is nothing inherently wrong with females adhering to femininity and males adhering to masculinity, it is likely that doing so will limit the behaviors they display. Therefore, as Martin et al. (2017) states,

“[b]ecause of these role restrictions, from Bem's (1975) perspective, being gender traditional was equated with rigidity; being androgynous (non-traditional) was equated with flexibility, and flexibility was related to adaptive and positive mental health” (Martin et al., 2017, p. 5–93).

Therefore, presenting female protagonists who display stereotypical behavioral profiles could encourage the young girls who are engaging with them to display predominantly feminine behaviors themselves, and indeed, there is evidence to suggest that this is the case (Wohlwend, 2012; Coyne et al., 2016; Golden and Jacoby, 2018). This could have implications for their adaptability and mental health (Bem and Lewis, 1975 as cited by Martin et al., 2017).

The current research has also helped establish that the portrayal of men in Disney animations is not stereotypical, which supports the previous quantitative content analyses (England et al., 2011; Hine et al., 2018a). In line with the argument made above, this may be beneficial to young boys as being presented with less limited male behavioral profiles may encourage them to be less restricted in their own behavior (Martin et al., 2017). Indeed, it seems that exposure to feminine behavioral profiles may be positive for young boys who seem to display more diverse toy preferences and behavioral profiles as a result (Coyne et al., 2021).

The question that remains is *why* the Disney corporation is consistently presenting stereotyped female protagonists, and non-stereotyped male protagonists. Is it that they believe that by presenting feminine behavioral profiles they will more efficiently capture a female audience? Or is it because, as Davis (2013) states:

“Disney is aware that it cannot be too radical with its depictions and themes: while controversial topics may be fine in some genres, they tend to be problematic in the family and children's film markets. It is not necessarily, as some would have it, that Disney “promotes” conservative ideas; rather, long experience has taught them to be careful with their level of experimentation. Go too far, and they lose the audience, lose money, and have to deal with a film which becomes a drain on the studio's resources” (Davis, 2013, p. 251).

Perhaps then, the long-standing success of films produced by Walt Disney Animation Studios which have historically, according to this work, presented relatively consistent gender profiles, justifies their (re)production of protagonists with similar gendered traits. In this sense, the producers may be restricted by Disney's previous legacy of hugely successful animations which span over 80 years. Arguably, the Walt Disney Animation Studio may wish not to experiment with portraying gendered profiles that are far from those of its previously successful protagonists, because as Davis (2013) states, it may prioritize commercial success over producing animated feature length films with entirely progressive (gender) ideals. Maybe a more complete examination of the company's content is necessary, including its recently acquired Marvel and Star Wars franchises, to more completely understand the company's gendered representations. Perhaps its acquirement of pre-existing franchises is its attempt to provide more progressive gendered representations outside of the formula it has established within its own animated feature length films. Further research will be necessary in order to comment.

## Data availability statement

The original contributions presented in the study are included in the article/Supplementary material, further inquiries can be directed to the corresponding authors.

## Author contributions

LC: Conceptualization, Data curation, Formal analysis, Investigation, Methodology, Project administration, Resources, Supervision, Validation, Visualization, Writing – original draft, Writing – review & editing. BH: Conceptualization, Methodology, Resources, Visualization, Writing – review & editing. DE: Conceptualization, Methodology, Resources, Writing – review & editing. PF: Conceptualization, Investigation, Methodology, Validation, Visualization, Writing – review & editing. RA: Data curation, Investigation, Validation, Writing – review & editing. SJ: Data curation, Investigation, Validation, Writing – review & editing. MG: Data curation, Investigation, Validation, Writing – review & editing.
