# Supplementary material for: The gendered behaviors displayed by Disney protagonists
Source: Front Sociol. 2024 May 6;9:1338900. doi: 10.3389/fsoc.2024.1338900 (PMC11102966; doi:10.3389/fsoc.2024.1338900)
Supplement: Supplementary file 1 [file Data_Sheet_1.PDF]

frontiers

Supplementary Material

The Gendered Behaviours Displayed by Disney Protagonists

Lucy Clarke[1*], Benjamin Hine[2*], Dawn England[3], Poppy P. M. S. Flew[4], Ritaj Alzahri[5], Stepheni N. Juriamsz[6], Ma. J. B. C. Garcia[7].

*** Correspondence:** Corresponding Author: Lucy.Clarke@uwl.ac.uk

Supplementary Material 1: The Coding Framework

1. *Focuses on physical appearance*- adjusting physical appearance for the purpose of making it look better or to draw attention to it. Also coded when a character comments on, is distracted by or admires their own appearance.

2. *Described as physically attractive*- either described as beautiful or handsome by other characters.

3. *Confident*- coded if a character explicitly says they are confident/competent in their abilities or comes across as cocky/overconfident. Also coded if a character suggests they can help in a problematic situation.

4. *Lacks confidence*- coded when a character doubts themselves or seems to think they are incapable or not worthy of something. Also coded if a character seems embarrassed or shy.

5. *Seeks advice or approval*- wanting, directly asking for, or accepting (without having asked for) emotional or mental support. Also coded if they wanted reassurance from another character.

6. *Assertive towards adults*- insistence upon a right or claim, the action of declaring or positively stating or making a demand of someone. Assertiveness included polite assertiveness with a hint of aggression. Assertiveness was a strong, direct assertion of a position or idea.

7. *Assertive towards children/animals*- insistence upon a right or claim, the action of declaring or positively stating to children or animals only. Assertiveness included polite assertiveness with a hint of aggression. Assertiveness was a strong, direct assertion of a position or idea.

8. *Submissive*— yielding to power or authority, humble and ready obedience. This trait was usually in response to another character's assertiveness. Includes subservience- unquestioningly obeying orders even when they are unreasonable and being controlled.

9. *Knowledgeable and experienced*- showing that they have knowledge/intelligence or life experience lacked by others. For example, when a character tells a story to another for the purpose of teaching them or warning them based on their previous experiences or knowledge.

Also coded when a character displays a specific skill with ease or outsmarts another character.

10. *Needs teaching*- coded when a character asks to be taught a physical skill or says they want to learn about something they have no experience of. Also coded if another seems surprised that the character does not know/has no experience of something or if a character is tricked or manipulated by another and is naïve.

11. *Brave*—courageous, daring, intrepid, heroic. Bravery often involved leadership in the face of danger. Can also be coded if a character performs a rescue or intervenes in a situation with the intent of protecting another from harm even when putting themselves in danger by doing so, or when vocalising they wish to protect another character.

12. *Scared*- frightened in a current situation (rather than being apprehensive about an upcoming event which would be coded as cautious) or is concerned about others. Can be coded when a character seems anxious.

13. *Leader*—one who leads, inspires a group of people, or brings them together, acts as a commander. Leader was only coded if the character was leading a group of people, not animals and not just him or herself. It also was only used to describe physical leadership in which a person is seen in front of and directing people and involved giving orders.

14. *Nurturing*- to care for and encourage the growth or development of, to foster. Being nurturing required direct interaction and was often shown as mothering. It involved prolonged touching and attention in a soothing manner (different than a brief instance of affection) or lending care in a loving way to either animals or people.

15. *Helpful*- rendering or affording physical assistance when needed, requiring an action that gave another person or animal direct assistance in a harmless/non-rescue situation. It was not used in a broader way to describe a character's role in a scene. Also coded if a character supports another by offering advice.

16. *Victimised/helpless*- a character needed assistance of another character to get out of a situation/needed to be rescued. Coded when someone suffers severely in body or property through cruel or oppressive treatment that they cannot escape. May suffer physical harm or suffer abuse. Can also be coded if a character is being manhandled.

17. *Shows affection*- towards a person or animal. A display of love such as a hug, a kiss, or an individual touch for the point of illustrating affection.

18. *Physically strong*- hitting or moving something, providing evidence that the character had a strong physical effect on the person or object. Also coded when a character climbs something that requires strength. This was different from a simple athletic display. There was a separate code for athletic, defined below, and the codes were mutually exclusive, as it was understood that displays of physical strength often incorporated some athleticism.

19. *Physically weak*—not being able to succeed in something that takes physical strength. It was often accompanied by needing help or else failing. Also coded when a character falls.

20. *Athletic*—a specific action such as a jump or kick that was large enough to require some athleticism. Running was also coded as athletic.

21. *Seeks adventure*- wants to search for, to investigate, to want to find out or explore the unknown. Coded when a character questions their life/situation or longs for a change. May express they 'want more' from their life when a character feels like something better must exist.

22. *Cautious (of adventure*)- weighing up the right thing to do when presented with the prospect of adventure. Coded when a character expresses the potentially negative consequences of their actions. Also coded when a character is hesitant or expresses that they do not know what to do.

23. *Hopeless*- becoming disheartened, wanting to give up on something such as a mission, pursuit, or person. The character may appear to be in state of despair, for example, their face may be in their hands, they may be physically slumped, or their body language/facial expression may suggest they are giving or have given up. Can also be coded if they express their readiness to give up vocally.

24. *Independent*- doing something despite being advised not to or it is against the norm. Performing an independent action against many, being alone when it was not the norm, or not participating in the expected culture. Not depending on the authority of another. Is autonomous/self-governing.

25. *Daydreaming*- being lost in thought or appearing distracted by their own thoughts. May include singing, humming, twirling, while engaged in thought as if in their own world.

26. *Wants to find romantic love*- explicitly expressing the desire to find love or marry generally, or after having met and fallen in love with another character.

27. *Uninterested in love*- character expresses that they do not want to find a partner, seems oblivious to the opposite sex or in extreme cases, expresses disgust at idea of finding love.

28. *Shows romantic interest*- coded when a character encourages conversation with the intention of getting to know another character in a way that suggests a romantic interest is being or will be developed. Can also be coded when a character is unable to control themselves when their love interest is around, they may be glazed over or look amazed, mesmerised, or stunned. They may lose control over their body or struggle to speak.

29. *Purposefully troublesome*- causing trouble, turmoil, disturbance for entertainment including in cheeky manner or to gain something, including stealing. Also coded when the character was being discussed by other characters in a way that made clear that the character had deliberately caused trouble that needed to be solved or needed to be reprimanded for their actions.

30. *Incompetent/unintentionally troublesome*- unintentionally getting themselves and/or others into a troublesome or dangerous situation. Also coded when the character was being discussed by other characters in a way that made clear that they caused trouble due to incompetence. Also coded when a character is clumsy.

31. *Sensitive/empathetic* —perception, knowledge, and understanding of other people's/ animals' emotions. Being able to understand how another character feels. Can be coded when a character listens to another's problems or emotionally supports another character. Can also be coded when a character does something nice for another, and/or is selfless, friendly, and warm.

32. *Insensitive*- unaware of another person's emotions or does not care about them. Could also be coded when a character does not show emotion in a sad situation such as a death.

33. *Angry/frustrated* – an outburst of anger in which the character may shout, scream, or otherwise express their frustration vocally or physically. Also coded when a character goes red in the face when provoked or when a character storms away from another character or situation.

34. *Panic*- a sudden uncontrollable fear or anxiety, often causing irrational or unthinking behaviour.

35. *Calm*- able to keep level-headed while others may be expressing anger. Could be coded when a character is calm when anger would be an acceptable response. Coded if the character is acting as a mediator in an argument or can think clearly in a stressful situation.

36. *Physically aggressive*- smashing/ throwing objects or being physically violent towards others when this is not a result of anger frustration. Can also be coded for less extreme aggression such a grabbing (a character, their clothing, or an object) as well as snatching something from another character.

37. *Verbally aggressive*- making threats, or verbally attacking another character for purpose of upsetting or intimidating them.

38. *Ashamed/guilty*— affected with shame, the painful emotion arising from the consciousness of dishonouring and guilt.

39. *Sad*- coded when a character has tears in their eyes, shedding a single tear or some tears but is not hysterical when in a sad situation, or because of upset. Was also coded if a character's facial expression or body language suggests they are unhappy.

40. *Collapses crying*—the character puts his/her face down, such that it was no longer visible, and cries, (hysterically) usually in rocking shakes and sobs. Sitting and crying while showing the face did not count; the character must have thrown him/herself on or against something (e.g., a bed, the floor).

41. *Excited/amazed*- a character may express their excitement vocally by suggesting that they are looking forward to an event or situation. They may physically express excitement and this can be coded when a character is jumping up and down or clapping out of eagerness, or their eyes widen when looking at something. Was also be coded if a character become hyperactive and rushes around or speaks quickly due to excitement.

42. *Expresses positive emotions (excluding excitement)*- a character may vocalise that they are happy or content. This may be reflected in their facial expressions or body language. They may also express their happiness for another character.

43. *Unimpressed*- being unamused, having no interest in something or underreacting to something that is expected to bring excitement, may seem underwhelmed/bored.

44. *Charming*- when a character tries to or succeeds in being liked and/or trusted by delighting, attracting, or fascinating another character.

45. *Deceitful*- coded when a character deliberately lies about something (including a small unimportant lie) or is deemed untrustworthy. Also coded when a character creates a plot or executes a plan to bring down another character behind their back. Can also be coded when a character creates a distraction.

46. *Honest*- tells the truth even if it gets them in trouble. Coded when a character confesses something or says/behaves as though they do not want to lie.

47. *Shocked/confused*- coded when a character seemed to be confused or unable to comprehend something or is shocked. This could be reflected in facial expressions.

48. *Funny/playful*- when a character says or does something for the purpose of being entertaining or having fun including mocking another character or referring to them using a nickname in a light-hearted manner.

49. *Selfish*- does something for their own gain even if this is at the detriment of another character. Was also be coded if a character is being competitive.

50. *Domestic*- coded when a character is doing domestic work or chores within the home.

51. *Voluntarily singing or dancing*- coded when a character was willing to dance or sing or did so voluntarily or without persuasion.

52. *Reluctant to sing or dance*- coded when a character either refused to sing or dance or needed persuasion to do so. Also coded if a character says they do not want to sing or dance.

Supplementary Material 2: Gendered Split of the Coding Framework

Masculine Traits	Feminine Traits	Gender Neutral Traits
Shows romantic interest	Focuses on physical appearance	Deceitful
Seeks adventure	Cautious (of adventure)	Honest
Physically strong	Physically weak	Shocked/confused
Assertive towards adults	Assertive towards children/animals	Selfish
Insensitive/unempathetic	Sensitive/empathetic	Calm
Independent	Submissive	Hopeless
Athletic	Shows affection	
Knowledgeable/experienced	Needs teaching	
Physically aggressive	Helpful	
Verbally aggressive	Unintentionally troublesome	
Leader	Purposefully troublesome	
Confident	Lacks confidence	
Angry/frustrated	Ashamed/guilty	
Charming	Collapsed crying	
Uninterested in love	Wants to find romantic love	
Reluctant to sing/dance	Voluntarily singing/dancing	
Unimpressed	Domestic	
Funny/playful	Nurturing	
Brave	Scared	
	Victimised/helpless	
	Sad	
	Described as physically attractive	
	Panic	
	Daydreaming	
	Seeks advice/approval	
	Excited/amazed	
	Expresses positive emotions (excluding excitement)	
Total = 19	Total =27	Total=6
